# Klotho interferes with a novel FGF-signalling pathway and insulin/Igf-like signalling to improve longevity and stress resistance in *Caenorhabditis elegans*

**DOI:** 10.18632/aging.100195

**Published:** 2010-09-09

**Authors:** Marie-Thérèse Château, Caroline Araiz, Simon Descamps, Simon Galas

**Affiliations:** ^1^ University of Montpellier1, Faculty of Pharmacy and Pharmaceutical Sciences, B.P. 14491, F-34093 Montpellier Cedex 5, France; ^2^ University of Montpellier2, Faculty of Sciences, F34095 Montpellier Cedex 5, France; ^*^ CNRS, CRBM-UMR 5237, F-34293 Montpellier Cedex 5, France; ^3^ Present address: Institute of Healthy Ageing, Room 324, The Darwin Building University College London, London WC1E 6BT, UK

**Keywords:** C. elegans, Klotho, FGF signalling, insulin/Igf-like signalling, aging, stress resistance

## Abstract

Klotho exerts anti-aging properties in mammals in two different ways. While membrane-bound Klotho, which is primarily expressed in the kidney, acts as an obligate co-receptor of FGF23 to regulate phosphate homeostasis, secreted Klotho, resulting from the shedding of the KL1-KL2 ectodomain into the bloodstream, inhibits Insulin/IGF1 signalling. However, the underlying molecular mechanisms are not fully understood. Here, we investigated the biological role of Klotho inCaenorhabditis elegans.

Two redundant homologues of the *klotho* gene exist *in C. elegans* and encode predicted proteins homologous to the β glucosidase-like KL1 domain of mammalian Klotho. We have used a genetic approach to investigate the functional activity of Klotho in *C. elegans*. Here, we report that whereas Klotho requires EGL-15 (FGFR) and EGL-17 to promote longevity and oxidative stress resistance, it is not involved in the regulation of fluid homeostasis, controlled by LET-756. Besides revealing a new post-developmental role for EGL-17, our data suggest that the KL1 form of Klotho is involved in FGF23-independent FGF signalling. We also report a genetic interaction between Klotho and the DAF-2 (Ins/IGF1R)/DAF-16 (FOXO) pathway. While the regulation of longevity requires functional DAF-2/DAF-16 signalling, the control of oxidative stress resistance involves a DAF-2- independent, DAF-16-dependent pathway, suggesting that Klotho may target either DAF-2 or DAF-16, depending of environmental conditions. Thus, the predictive KL1 form of Klotho appears to crosstalk with both FGF and Insulin/IGF1/FOXO pathways to exert anti-aging properties in *C. elegans*.

## INTRODUCTION

Although the *klotho* gene was first reported to retard aging in mice [1,2], human *Klotho* gene poly-morphisms have been significantly linked with reduced longevity [3]. The human *Klotho* gene encodes either a type IItransmembrane protein, consisting of two extracellular β-glucosidase-like domains (KL1 and KL2), or a putative secreted form, resulting from alternative splicing that leads to the production of a single KL1 domain [4]. In addition, both full KL1-KL2 extracellular domain (130 kDa) and/or C-terminal KL1 (68 kDa) domain have been reported to be released from the membrane-bound isoform by proteolytic cleavage that involves ADAM10 and ADAM17 proteases [5]. While the membrane-bound Klotho isoform is mainly expressed in distal convoluted tubules of the kidney, where phosphate reabsorption occurs, the entire KL1-KL2 extracellular domain has been detected as a 130 kDa polypeptide in the cerebrospinal fluid, blood, and urine [6,2]. Therefore, this 130 kDa Klotho polypeptide has been referred as the secreted form of Klotho [7].

In human cells, the alternative transcript, encoding for the KL1 domain, predominates over the full length transcript encoding for the membrane-bound isoform of Klotho [4]. The KL1 secreted isoform has not yet been documented, possibly on account of a lack of KL1 secreted isoform detection in body fluids.

After a decade of extensive studies in mammals, different Klotho isoforms appear to play distinct functions. The membrane-bound isoform acts as an obligate co-receptor for FGF23 [8,9]by interacting with Fibroblast Growth Factor Receptors (FGFRs) in the kidney. FGF23 is a bone-derived hormone that inhibits both phosphate reabsorption as well as vitamin D biosynthesis. Interestingly, mice lacking either Klotho or FGF23 exhibit a premature-aging syndrome [10], which reveals an unexpected link between phosphate metabolism and aging (reviewed in [7]). The secreted Klotho acts as an endocrine regulator of several cell surface glycoproteins, including ion channels or growth factor receptors, such as the insulin/Igf-like receptors. Moreover, the latter have been involved in aging control and stress resistance (reviewed in [11,12]). Whereas the putative sialidase activity of the secreted Klotho may inhibit the internalization of calcium TRPV5 [13] and potassium ROMK1 [14] channels, the mechanism by which the secreted Klotho isoform inhibits Ins/Igf-like receptor activity remains to be determined. Thus, the effects of both Klotho isoforms converge towards a positive modulation of lifespan, but the underlying molecular mechanisms are far from being understood.

A *C. elegans* lifespan extension can be achieved by reduced Daf-2/insulin/Igf-like signalling [15,16]. Furthermore, lifespan modulation effects have been also reported in worm [17,18,19], fly [20,21] and mice [22,23] upon downregulation of the conserved insulin/Igf-like pathway.

A single FGFR (EGL-15) and two FGF ligands (LET-756 and EGL-17) have been identified in *C. elegans*. While LET-756 plays an essential function in regulating fluid balance [24], EGL-17 is involved in sex myoblast (SM) migration [25]. Thus, two ligands can mediate specific functions depending on tissue-specific receptor isoform expression. EGL-15(5A) is predominantly expressed in the worm M cell lineage and is necessary for the gonadal chemoattraction of the migrating SM [25] while EGL-15(5B), which is principally localized in the hypodermis, is required for viability [26,27]. However, despite specific phenotypes that have been already reported, some redundant functions have been reported for both LET-756 and EGL-17. For instance, EGL-15 activation by either LET-756 or EGL-17 is involved in protein degradation in *C. elegans* muscle [28].

Here, we report that two Klotho gene homologues exist in the nematode. In addition, we present genetic evidence for EGL-15 (FGFR) requirement by Klotho to regulate longevity and oxidative stress resistance in *C. elegans*.

In addition, we also examined FGFs ligands in *C. elegans* and found that between the two known EGL-15 ligands, only EGL-17 (that is homologous to the mammalian FGF8 subfamily) appears necessary and sufficient to induce Klotho signalling. Besides the report of a new post-developmental and specific role for EGl-17 in the regulation of both aging and oxidative stress resistance, our results reveal an unexpected link between Klotho and a novel, FGF23-independent, FGF-signalling pathway. We also report a genetic interaction between Klotho and the Daf-2/insulin/Igf-like signalling pathway.

While lifespan regulation requires a functional Daf-2/insulin/Igf-like signalling cascade, the control of oxidative stress resistance involves both a DAF-2- independent and a DAF-16-dependent pathway, suggesting that Klotho may target either DAF-2 or DAF-16, depending on environmental cues. Taken together, our findings strongly indicate that a predicted KL1 isoform of Klotho requires the FGF signalling pathway to crosstalk with the Daf-2/insulin/Igf-like pathway, in order to regulate aging and oxidative stress resistance, in *C. elegans*.

## RESULTS

### 1- Genetic characterization of the two *klotho* genes in *C. elegans*

**Figure 1.**
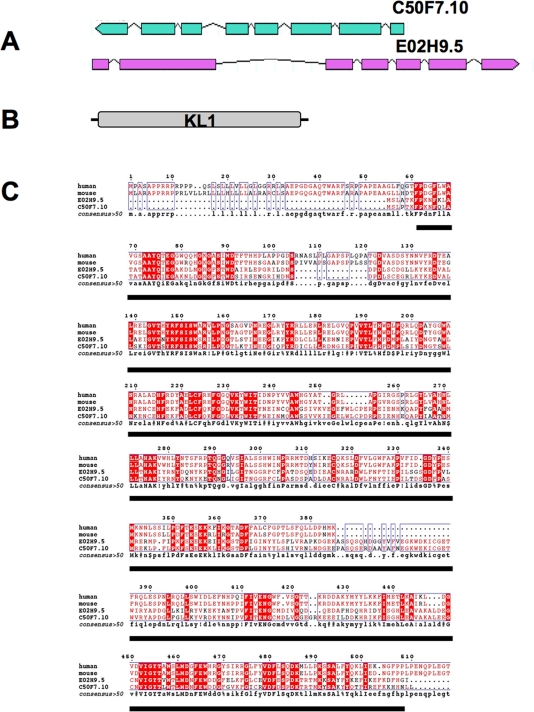
Characterization of Klotho in *C. elegans*. (**A**) Genomic organization of both *C. elegans* C50F7.10 (1,95 kb) and E02H9.5 (2,3 kb) genes, localized on chromosome IV and III, respectively. Coding regions are indicated by boxes, and introns are represented as lines. The corresponding ORFs share similar size (about 1,44 kb) and are organized in 8 and 7 exons for C50F7.10 and E02H9.5, respectively. (**B**) The predictive molecular organization of either C50F7.10 or E02H9.5 gene products essentially consists in a sole b-glucosidase-like KL1 domain. Note that a KL1 form of Klotho may be expressed either by differential splicing or post- translational cleavage, in mammals. (**C**) Alignment of alternatively spliced forms of human and mouse Klotho compared to both *C. elegans* C50F7.10 and E02H9.5 gene products, identified in the WormBase bank as WP: CE 04248 and WP: CE 09122, respectively. Identical amino acid residues are highlighted. The conserved KL1 domain is underlined. Alignment was performed using the ESPript program [60].

The *C. elegans* genome contains two sequences homologous to the mammals *klotho* gene : C50F7.10 and E02H9.5. Although these two paralogues differ in their genomic organization (Figure 1A), they encode ORFs of similar size and appear genetically redundant, since no obvious function emerged from previous mass RNA-mediated gene interference (RNAi) assays [29]. In addition, a sole putative *klotho* gene has been predicted in the *C. briggsae* genome [30]. Considering that the latter species diverged from *C. elegans* ~100 millions years ago, it is probable that a unique gene has been retained by evolution.

The predictive sequences of the C50F7.10 and E02H9.5 gene products (479 and 475 amino-acids, respectively) are 76% identical and share 32 to 34% amino acid identity with their mammalian counterparts. Interestingly, both *C. elegans* predicted gene products consist of a sole β-glucosidase-like domain that is homologous to the highly conserved KL1 domain of human and mouse Klotho proteins but that lacks a secretory signal peptide (Figure 1B,C). However, a potential secretion mechanism, via an endoplasmic reticulum/Golgi complex-independent pathway, cannot be excluded. Such a mechanism has been described in mammals for some secreted FGFs [31,32]. Furthermore, the membrane-bound Klotho could not be observed at the surface of kidney cells in mammals, but only as a diffuse expression in the cytoplasm. In addition, a combination of both conventional and a novel, Klotho-dependent, secretory pathways has also been reported [33,34].

**Figure 2.**
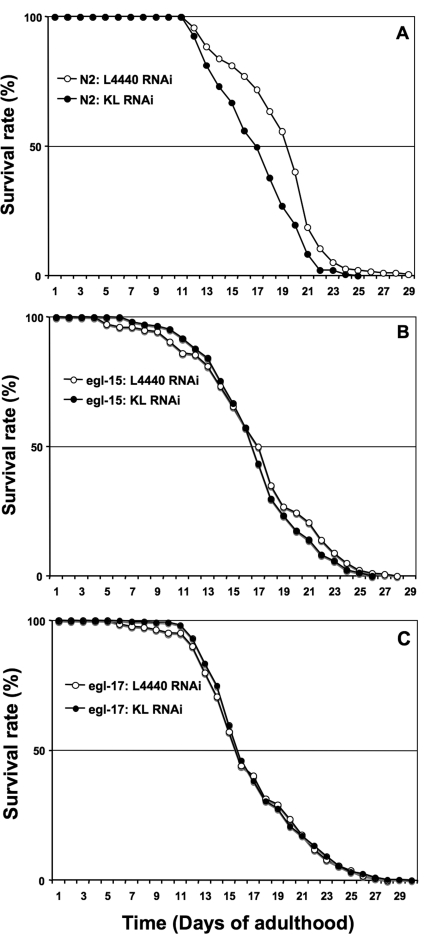
A genetic interaction between Klotho and the EGL-17/EGL-15 (FGFR) signalling pathway can positively modulate lifespan. Adult worm lifespan analysis was performed at 20°C on RNAi plates as described in the text. Animals of indicated genotype were submitted to either klotho RNAi (solid symbol) or control L4440 vector RNAi (open symbol) throughout adulthood. See Table 1 for corresponding quantitative data and statistical analysis.

### 2- Klotho requires the EGL-17/EGL-15 (FGF-R) complex to regulate *C. elegans* longevity

To analyse the *in vivo* function of Klotho in *C. elegans*, we have chosen a genetic approach by using RNA-mediated gene interference (RNAi). To circumvent the genetic redundancy of C50F7.10 and E02H9.5, we designed RNAi capable of simultaneously reducing the expression of both paralogues.

As previously reported in mammals [1], we then assessed whether *klotho* gene knockdown can affect worm lifespan. As expected, N2 (wild type) worms showed a highly significant reduction (LogRank p < 0.0001) of longevity when submitted to *klotho* RNAi, in comparison with control (Figure 2A; Table 1). Similar results were observed when the E02H9.5 (ok1830) knockout strain was tested in place of the N2 (wild type) strain, supporting the genetic redundancy of the two paralogues (not shown).

In mammalian cells, the membrane-bound Klotho isoform has been reported to bind to multiple FGFRs, increasing their affinity to FGF23. Because of its weak affinity to FGF23, the secreted Klotho is not likely to function as a soluble co-receptor for FGF23. Whether this secreted Klotho product can modulate lifespan by interacting with FGF signalling in a FGF23 independent manner has not been investigated.

**Table 1.** Life span modulation by klotho requires the EGL-17/EGL-15 (FGFR) signalling pathway

Strains	RNAi	50th percentile^a^	Mean lifespan days (+/-SD)	Max lifespan	Code^b^	Statistics^c^ (Log-Rank)	N (Censored)^d^
N2 (wt)						p < 0.0001(B)	
						p < 0.0001(C)	
	Control	19	18.04(0.25)	29	A	p < 0.0001(D)	192(19)
						p = 0.001(E)	
						p = 0.000(F)	

						p < 0.0001(A)	
						p = 0.037(C)	
	*klotho*	16	16.16(0.23)	24	B	p = 0.54(D)	193(6)
						p = 0.235(E)	
						p = 0.261(F)	

*egl-15 (n484)*						p = 0.189(D)	
						p < 0.0001(A)	
	Control	17	16.07(0.35)	27	C	p = 0.037(B)	180(27)
						p = 0.568(E)	
						p = 0.665(F)	

						p = 0.189(C)	
						p < 0.0001(A)	
	*klotho*	16	15.97(0.29)	25	D	p = 0.54(B)	171(32)
						p = 0.173(E)	
						p = 0.204(F)	

*egl-17 (n1377)*						p = 0.768(F)	
						p = 0.001(A)	
	Control	15	16.04(0.28)	27	E	p = 0.235(B)	229(49)
						p = 0.568(C)	
						p = 0.173(D)	

						p = 0.768(E)	
						p = 0.000(A)	
	*klotho*	15	16.18(0.24)	29	F	p = 0.261(B)	283(51)
						p = 0.665(C)	
						p = 0.204(D)	

The tabulated data show the average results of four to eight independent RNAi trials at 20°C. Worms of indicated genotypes were assayed for life span by feeding on E. coli HT115 strain bacteria producing either control or klotho RNAi (see Material and Methods). XLSTAT-life statistical software (Addinsoft, New York, NY, USA) was used to plot survival data by the Kaplan Meier method and differences between survival curves calculated using the Log-Rank test with 95% confidence.

(a) Represents the 50th percentile (the age when the survival fraction of animals reaches 0. 50).

(b) Experiment identification code.

(c) Probability of being identical to other life span experiment given in parentheses.

(d) Total death scored (number of censored values).

We then asked whether FGF signalling is a determinant in the regulation of worm lifespan by Klotho. To avoid possible redundant effects of the two EGL-15 ligands, we first tested whether *klotho* gene deficiency alters lifespan in an *egl-15* gene-dependent manner.

As shown in Figure 2B and Table 1, the mean life-span of the *egl-15* (n484) reduction-of-function allele, fed with either control or *klotho* RNAi, was clearly identical to N2 (wild-type) worms fed with *klotho* RNAi only (LogRank p = 0.037; p = 0.54, respectively).

Downregulation of both *egl-15* and *klotho* genes reduces lifespan by a similar order of magnitude, strongly suggesting that the entire modulation of lifespan by *klotho* gene in worms, is mediated by the FGFR EGL-15. Interestingly, *egl-15* (n484) has been reported to mimic the phenotype of an *egl-17* reduction-of-function allele [35]. On the basis of this mutant allele effect, we then assessed whether *klotho* gene deficiency might alter lifespan in an *egl-17* gene-dependent mechanism. As expected (Figure 2C; Table 1), the mean lifespan of the egl-17 (n1377) reduction-of-function allele, fed with either control or *klotho* RNAi, was comparable to that of N2 (wild-type) worms fed with *Klotho* RNAi only (LogRank p = 0.235; p = 0.261, respectively). Since either reduction-of-function of *egl-17* or *klotho* gene knock down can impair worm lifespan to a similar degree, our data indicate that EGL-17 alone (of the EGL-15 ligands) is necessary and sufficient to modulate lifespan in a Klotho-dependent manner. Moreover, we demonstrate, for the first time, a specific involvement of EGL-17 for normal post-developmental events in worm. Taken together, our results support genetic evidence that Klotho can signal *via* the EGL-17/EGL-15 pathway to regulate worm lifespan in physiological conditions.

Of the two known FGFR ligands in worm, LET-756 has been primarily implicated in fluid balance regulation [24], and more recently in muscle protein degradation [28]. However, because of the essential function accomplished by LET-756, we were not able to assess its implication in the regulation of lifespan, but we cannot exclude that it does not also play a role. Moreover, according to our results, such a potential role should be independent of Klotho.

### 3- Klotho is not involved in the worm fluid balance homeostasis

In *C. elegans*, the vital maintenance of fluid homeostasis is under the control of the LET-756/EGL-15 (B) complex which activates the LET-60 (RAS) /MPK-1 (MAPK) pathway [36]. Such a regulatory pathway is reminiscent of the role of FGF23 in the kidney, where it regulates ionic homeostasis and, therefore, helps in the maintenance of global fluid balance. In fact, the mammalian FGF-23/Klotho /FGFR-1 complex has been reported to activate the MAP kinase pathway [8].

We then assessed for a possible link between Klotho and functions implicating LET-756/EGL-15. In *C. elegans*, the existence of a negative regulator of FGFR signalling allows genetic activation of EGL-15. EGL-15 (FGFR) signalling is normally attenuated by a receptor tyrosine phosphatase, encoded by the *clr-1* gene. Reduced CLR-1 receptor activity provokes an over-activation of EGL-15 (FGFR) signalling leading to a Clr (clear) phenotype, characterized by fluid accumulation within the pseudo-coelom [37]. Thus, *clr-1* mutants have proven useful for screening Soc (suppressor of Clr) regulators, which can reverse the Clr phenotype by decreasing EGL-15 signalling [35,36].

We took advantage of the Clr model phenotype described above to assess whether *klotho* gene deficiency might diminish EGL-15 signalling in a *clr-1* genetic background and thus suppress, at least partially, the Clr phenotype.

When temperature-sensitive allele *clr-1(e1745)* worms are shifted to restrictive temperature (25°C), they develop a Clr phenotype. We then pre-induced *clr-1(e1745)* worms to either *klotho* or control RNAi at permissive temperature (15°C) before shifting them to the restrictive temperature.

**Figure 3.**
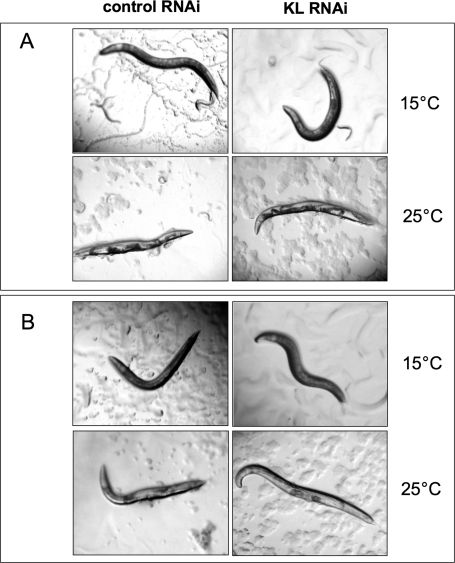
Klotho gene knockdown cannot suppress the Clr phenotype induced by genetic activation of EGL-15. Adult worms were pre-induced with either *klotho* or control RNAi at permissive temperature (15°C), prior to being either maintained at 15°C or shifted to restrictive temperature (25°C) which allows the development of a Clr phenotype. (**A**) Experiments were conducted in a *clr-1(e1745)* genetic background. The induced Clr phenotype is characterized by both intestine and gonad floating in an enlarged fluid-filled pseudocoelomic cavity. (**B**) Controls, performed in a *clr-1(e1745); let-756(s2613)* reduction-of-function double mutant show a partial suppression of Clr phenotype, independent of the *klotho* gene status.

As shown in Figure 3A, *klotho* knockdown failed to suppress the Clr phenotype, characterized by both intestine and gonad floating in an enlarged fluid-filled pseudocoelomic cavity. As controls, similar experiments were done using a *clr-1(e1745); let-756(s2613)* reduction-of-function double mutant in place of *clr-1(e1745)* mutant allele. In accordance with Borland's data [36], our results show that *let-756* gene activity reduction leads to a partial suppression of the Clr phenotype (Figure 3B). However, this latter observation appears to be independent of the *klotho* gene status.

Taken together, our data suggest that Klotho is not involved in the maintenance of fluid balance in the nematode. Furthermore, our observations again argue for a lack of interaction between Klotho and the LET-756 ligand.

### 4- Klotho targets the C. elegans Insulin/Igf-like/Daf-2 receptor and requires Daf-2/Daf-16 signalling pathway for lifespan modulation

**Figure 4.**
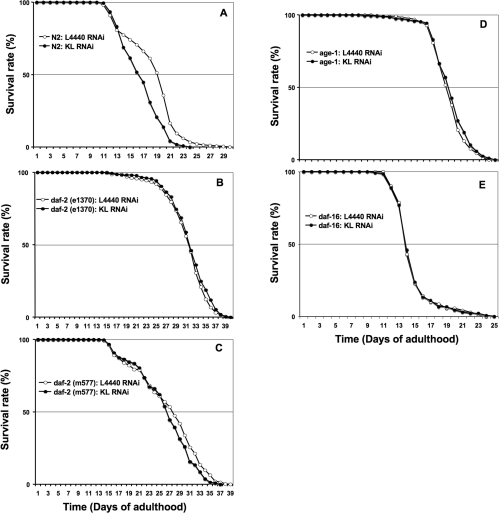
Klotho targets *daf-2* gene activity and requires a functional *daf-2/daf-16* genetic pathway for lifespan modulation. See Figure 2 for legend and Table 2 for corresponding quantitative and statistical analysis.

Overexpression of Klotho in mice has been reported to inhibit the activation of the Insulin/Igf-like receptor [2]. Furthermore, loss-of-function of the worm *daf-2* gene, that encodes for an Insulin/Igf-like receptor, can double worm lifespan [38]. We therefore asked whether Klotho behaves in the same way, by acting as a constitutive Insulin/Igf-likesignalling pathway repressor, in worm. With this in mind, we assessed whether *klotho* gene deficiency can modulate worm lifespan in a *daf-2* gene-dependent manner. Statistical analysis (Table 2) showed that *klotho* knockdown failed to induce detectable lifespan modulation (LogRank p = 0.027) in a *daf-2 (e1370)* genetic background (Figure 4B). However, we detected (Table 2) a slightly significant lifespan modulation (LogRank p = 0.002) when *klotho* knockdown was assessed in a *daf-2 (m577)* genetic background (Figure 4C). One possible explanation for such a *Klotho* RNAi effect discrepancy between the two *daf-2* reduction-of-function alleles assessed for lifespan could be differences in their *daf-2* mutant class. Thus, while *daf-2*(*e1370)* belongs to the *daf-2* class 2-allele and behaves as a kinase-dead-like mutant, the *daf-2* (*m577)* reduction-of-function allele, which belongs to the *daf-2* class 1-allele, is modified by a point-mutation in the ligand-binding site and therefore may exhibit residual, ligand-independent, catalytic activity [39,40]. Taken together, our data argue for *in vivo* lifespan modulation by Klotho mediated by the Insulin/Igf-like/Daf-2 receptor activity by either a ligand-dependent or -independent effect.

**Table 2.** Klotho interacts with the *daf-2/daf-16* genetic pathway for lifespan modulation. See Table 1 for legend.

Strains	RNAi	50th percentile^a^	Mean lifespan days (+/-SD)	Max lifespan	Code^b^	Statistics^c^ (Log-Rank)	N (Censored)^d^
N2 (wt)						p < 0.0001(H)	
						p < 0.0001(I)	266(23)
	Control	19	17.48(0.24)	29	G	p < 0.0001(J)	

						p < 0.0001(G)	
						p = 0.015(I)	216(11)
	*klotho*	16	15.76(0.20)	23	H	p = 0.001(J)	

*daf-16 (mgDf50)*						p = 0.996(J)	
						p < 0.0001(G)	70(3)
	Control	14	14.86(0.29)	24	I	p = 0.015(H)	

						p = 0.996(I)	
						p < 0.0001(G)	135(5)
	*klotho*	14	14.78(0.20)	25	J	p = 0.001(H)	

*age-1 (mg44)*	Control	24	23.59(0.19)	30	K	p = 0.077(L)	223(13)

	*klotho*	24	23.87(0.16)	31	L	p = 0.077(K)	358(7)

*daf-2 (e1370)*	Control	31	30.27(0.19)	39	M	p = 0.027(N)	527(22)

	*klotho*	31	30.81(0.16)	39	N	p = 0.077(M)	655(17)

*daf-2 (m577)*	Control	27	25.77(0.38)	38	O	p = 0.002(P)	286(29)

	*klotho*	26	25.19(0.24)	36	P	p = 0.002(O)	547(31)

In mammalian cell cultures, Klotho overexpression can diminish the recruitment of phosphatidyl-inositol 3-kinase (PI3-kinase) regulatory subunits that normally leads to Insulin/Igf-like receptor activation [2]. However, to date, the direct inhibition of PI3-kinase catalytic activity by Klotho has not been demonstrated. We investigated the involvement of PI3-kinase/AGE-1 in worms by submitting the strong loss-of-function allele *age-1(mg44)* mutant to *klotho* knockdown. As shown (Figure 4D; Table 2) we cannot detect any significant (LogRank p = 0.077) lifespan variation when compared to control. Taken together, our results demonstrate a fully functional Insulin/Igf-like/PI3-kinase signalling pathway for worm lifespan modulation by Klotho.

The worm lifespan extension induced by different *daf-2* class-mutants is believed to occur by de-repression of the FOXO transcription factor family member DAF-16 [41]. *Daf-16* gene products are thought to be permanently under negative regulation by the *daf-2*/*age-1* genetic pathway. Therefore, strong *daf-16* loss-of-function alleles can suppress the long-lived phenotype of *daf-2* mutants. We thus submitted the *daf-16* (*mgDf50*) deletion mutant to *klotho* RNAi. As expected, worm lifespan was not affected (LogRank p = 0.996) in this genetic background (Figure 4E; Table 2). This result makes the *daf-16* gene a major output for *klotho* gene effect on worm lifespan modulation.

In summary our data demonstrate that Klotho can significantly convert an N2 (wild type) worm lifespan (Figure 4A; Table 2) to a shorter one. However, since this negative lifespan modulation vanished when *daf-2, age-1* or *daf-16* mutants were submitted to *klotho* knockdown, we propose that Klotho de-represses DAF-16 by constitutively down-regulating DAF-2. Therefore we provide here strong evidence for a direct link between the Insulin/Igf-like/PI3-kinase/FOXO signal-ling pathway and *klotho* gene action on lifespan modulation in a whole organism, which then argues for an evolutionary conserved mechanism.

**Figure 5.**
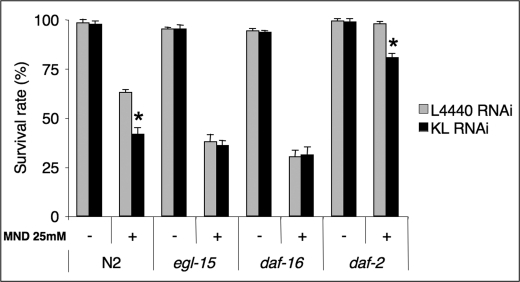
Klotho requires a functional EGL-17/EGL-15 signalling pathway to improve oxidative stress resistance by a *daf-16*-dependent but *daf-2*-independent genetic pathway. Adult worms of the indicated genotype were pre-induced to either *klotho* or control RNAi at 20°C, then subjected to oxidative treatment by 25mM Menadione during 72 h and their viability scored. Controls were performed in the absence of Menadione. Results are mean values +/- SD of at least four independent experiments. Statistical analysis was done by a Student t-test at *p < 0.05 signification level. At least 100 worms were scored for each test condition. All experiments were performed at 20°C.

### 5- Klotho requires functional EGL-17/EGL-15 signalling to improve oxidative stress resistance in a DAF-16-dependent and DAF-2-independent mechanisms

It has been reported that Klotho can increase oxidative stress resistance at the cellular and organism levels in mammals [42,43]. It is also believed that FOXO transcription factor de-repression by Klotho-induced downregulation of Insulin/Igf-like signalling should induce antioxidant enzymes overexpression.

Two antioxidant enzymes: manganese superoxide dismutase and catalase are known to facilitate removal of reactive oxygen species towards a permanent molecular scavenger action. Since increased resistance to oxidative stress has been associated with increased longevity in various species, including *C. elegans* [44], we then asked if Klotho may help protecting worms when subjected to a sub-lethal oxidative stress induced by Menadione [45].

As shown in Figure 5, both the *egl-15 (n484)* reduction-of-function allele and the *daf-16 (mgDf50)* deletion mutant, fed with either control or *klotho* RNAi, displayed an increased sensitivity to oxidative stress in similar fashion to the N2 (wild-type) worms fed with *klotho* RNAi only. As expected, similar results were obtained when the *egl-17(n1377)* reduction-of-function allele was used in place of the *egl-15(n484)* mutant (not shown).

These data suggest that Klotho requires functional EGL-17/EGL-15 signalling to improve oxidative stress resistance in a DAF-16-dependent manner. We further checked whether Klotho requires a fully functional DAF-2 tyrosine kinase domain to improve oxidative stress resistance. Surprisingly, the *daf-2(e1370)* loss-of-function allele showed an increased susceptibility to oxidation when fed with *klotho* RNAi with respect to control RNAi. Since the functional activity of the tyrosine kinase domain is impaired in this class mutant allele, our results strongly suggest that Klotho may activate DAF-16 by a different pathway from the canonical worm DAF-2/DAF-16 signalling cascade. Interestingly, some reports using mammalian models have shown possible FOXO activation by alternative pathways that involve inhibition of the serum- and glucocorticoid-inducible kinase SGK [46] or activation of either the c-Jun NH2-terminal kinase (JNK) [47] or β-catenin [48].

## DISCUSSION

Here, we report the first investigation of *klotho* gene function in a non-mammalian organism. The choice of the nematode *C. elegans* was motivated by the evolutionary conservation, from worm to human, of the signalling pathways controlling both longevity and oxidative stress resistance. The functional activity of Klotho in worms was investigated using a genetic approach, based on the simultaneous knockdown of both worm *klotho* paralogues, coupled to an epistatic analysis in relevant genetic backgrounds.

We first looked for a potential interaction between Klotho and FGF signalling, in *C. elegans*. Our results demonstrate that Klotho requires EGL-15, the single FGFR identified in worms, to regulate both lifespan and oxidative stress resistance. This suggests, at least in *C. elegans,* that the predicted KL1 isoform of Klotho may interact with the FGF pathway, possibly by targeting FGFR. Interestingly, N-glycosylation has been recently reported to negatively regulate EGL-15 activity *in vivo*[49]. According to our data, such a finding leads to the hypothesis that Klotho, *via* its putative sialidase activity, could de-repress EGL-15 by favouring the removal of inhibitory N-glycans.

Then, to assess the possible involvement of a specific EGL-15 ligand in Klotho signalling, three lines of evidence allowed us to discriminate between LET-756 and EGL-17: (i) the *egl-15 (n484)* reduction-of-function allele used here has been reported to mimic the phenotype of an egl-17 reduction-of-function allele [35]; (ii) similar results were obtained using the *egl-17 (n1377)* reduction-of-function allele in place of *egl-15 (n484)*; (iii) Klotho did not interfere with fluid balance regulation controlled by LET-756.

Taken together, our data demonstrate that the EGL-17 ligand is necessary and sufficient for Klotho signalling expression. Since the EGL-15(5A) isoform is specifically recognized by EGL-17 [26,27], a Klotho/EGL-17/EGL-15(5A) complex may positively regulate lifespan and oxidative stress resistance in worms.

Both phylogenetic and functional studies suggest that EGL-17 could be included, like mammalian FGF8, -17 and -18, in the FGF8 subfamily [50]. Interestingly, FGF8 may protect mammalian cultured neurones from oxidative stress [51]. Moreover, while a decrease in FGF8 signalling has been related to hypogonadotropic hypogonadism in human and mice [52], a similar syndrome was characterized in Klotho-deficient mice [11]. In *C. elegans*, EGL-17 is localized in the gonad where it functions as a chemo-attractant for the EGL-15(5A)-expressing SMs, during larval development [25,50]. By the new involvement of a possible Klotho/EGL-17/EGL-15(5A) complex in the control of both longevity and stress resistance, our results help to unravel a new post-developmental role for EGL-17 in *C. elegans*.

The secreted Klotho is already known to inhibit both insulin and IGF1-induced receptor autophosphorylation, when applied to cultured mammalian cells [2,53]. In accordance with these data, we report that Klotho requires a functional DAF-2 Insulin/Igf-like/Daf-16 (FOXO) pathway for positive modulation of worm lifespan.

We also demonstrate in a whole organism that Klotho may physiologically repress the tyrosine kinase activity of DAF-2 activated in either a ligand-dependent or -independent manner. Although the mammalian secreted Klotho is believed to behave independently of FGF signalling, the predictive KL1 isoform of Klotho requires EGL-17/EGL-15(5A) to regulate longevity in worm. Interestingly, a signal produced by the somatic gonad has been reported to lengthen worm lifespan by inhibiting DAF-2 (Insulin/Igf-like) activity [54]. Since the nature of this signal is still unknown, it is tempting to speculate that the putative Klotho/EGL-17/EGL-15(5A) complex, that is primarily localized in the gonad, could take part in the molecular mechanism of this yet unknown signalling.

The secreted Klotho has also been reported to suppress oxidative stress in mammals [42,43]. Indeed, the de-repression of FOXO consecutive to the inhibition of the Insulin/Igf-like signalling up-regulates the expression of antioxidant enzymes that are known to facilitate the removal of reactive oxygen species. Here, we report that Klotho, together with EGL-17/EGL-15(5A), improves worm oxidative stress resistance towards a DAF-16-dependent manner. In addition, by the use of a *daf-2 (e1370)* loss-of-function allele, we report that the protective effects of Klotho against a potent oxidative stress are independent of a functional DAF-2 receptor. The fact that C50F7.10 was found to be overexpressed in *daf-2* mutants [55] also supports this finding.

Our results strongly suggest that Klotho may activate DAF-16 by a pathway that is different from the canonical DAF-2/DAF-16 signalling cascade in worm.

**Figure 6.**
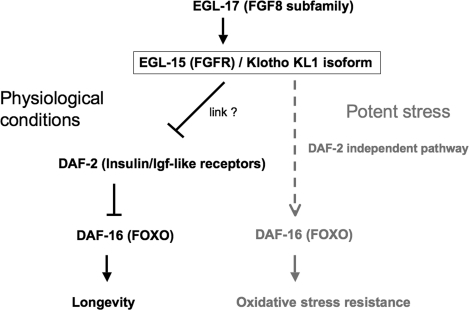
In adult worms the FGFR EGL-15(5A) targeted for activation by the Klotho KL1 isoform can allow EGL-17 ligand binding. Under physiological conditions, the Klotho/EGL-15/EGL-17 complex constitutively represses the DAF-2 (Insulin/Igf-like) receptors by a still unknown pathway. Such complexes may induce DAF-16 (FOXO) de-repression and subsequent overexpression of longevity factors, such as antioxidant enzymes. When worms have to cope with a potent stress, the Klotho/EGL-15/EGL-17 complex may directly activate DAF-16 by a DAF-2-independent pathway (dashed line). Such activation mechanism remains to be elucidated.

Interestingly, several studies have shown that FOXO can be activated by alternative pathways, involving the inhibition of the serum- and glucocorticoid-inducible kinase SGK [46] or the activation of, either the c-Jun NH2-terminal kinase (JNK) [47] or β-catenin [48].

In summary, our data suggest that Klotho could target either worm DAF-2 or DAF-16, depending of environ-mental cues. Indeed, Klotho seems to constitutively repress DAF-2 throughout lifespan while it could also directly activate DAF-16 when the nematode has to cope with intense stress (Figure 6). Since a functional EGL-17/EGL-15(5A) signalling is required in all cases, the predicted KL1 form of Klotho appears to link FGF and Insulin/Igf-like pathways, in *C. elegans*. How these two pathways can crosstalk remains to be determined. A potential intermediate could be the adaptor protein SHC-1, homologue of human p52Shc, that has been recently reported to modulate lifespan and stress response in *C. elegans*. Indeed, SHC-1 was shown to repress DAF-2 by an unknown mechanism and to activate DAF-16 by a JNK-involved pathway [56].

## EXPERIMENTAL PROCEDURES

### Strains and culture conditions

The following strains were used: Wild type Bristol (N2); E02H9.5 *(ok1830)*; MT1079 *egl-15(n484)*; MT3188 *egl-17(n1377)*; CB3241 *clr-1(e1745)*; PJ1153 *clr-1(e1745) [let-756(s2613) unc-32(e189) ccls55]*; CB1370 *daf-2(e1370)*; GR1032 *age-1(mg44)/mnC1[dpy-10(e128) unc-52(e444)]*; GR1307 *daf-16(mgDf50)* which were all provided by the *Caenorhabditis* Genetics Center (funded by the NIH National Center for Research Resources). DR1567 *daf-2(m577)* was kindly provided by David Gems, University College London, UK. Worms were maintained at 15°C and, unless specified, cultured at 20°C as described previously [57].

### Expression of RNAi constructs

E02H9.5 and C50F7.10 entire ORFs were cloned from *C. elegans* cDNA into a pBluescript II KS (+/-) vector (Stratagene, La Jolla, CA, USA). RNAi constructs were obtained after E02H9.5 and C50F7.10 ORFs were amplified from pBluescript II KS (+/-) and 1425 and 1464 bp respective fragments were inserted at the SmaI site of a pPD129.36 (L4440) vector (a generous gift of Johnathan Ewbank, CNRS, Marseille, France). RNAi producing bacteria were obtained as follows: after transformation with either pPD129.36, E02H9.5 or C50F7.10 RNAi construct, HT115 (DE3) strain bacteria were grown in LB medium in presence of both Ampicillin (50 μg/ml) and Tetracyclin (12,5 μg/ml) during 8 h, then seeded on NGM plates containing FUdR (98,5 μg/ml), Ampicillin (50 μg/ml) and IPTG (1mM) and induced overnight.

### RNAi feeding and lifespan assays

Synchronized L4 larvae fed with RNAi producing bacteria were allowed to grow at 20°C until death on supplemented NGM plates, including FUdr to avoid offspring [58]. Worms were examined every day for touch-provoked movement, dead worms were scored and worms that crawled off the plate or displayed extruded internal organs were censored. All lifespan assays were repeated at least three times. XLSTAT-life statistical software (Addinsoft, New York, NY, USA) was used to plot survival data by the Kaplan-Meier method and differences between survival curves were calculated using the LogRank test with 95% confidence.

### Real-time PCR

Synchronized N2 adult worms were fed with RNAi producing bacteria during 4 days at 20°C, total RNA was isolated and cDNAs were synthesized as previously described [45]. Real-time PCRs were performed in capillaries, using LightCycler Fast Start DNA Master^plus^ Sybr Green I kit (Roche Diagnostics, Meylan, France). Primers for the reference gene TBA2 are: Forward primer: 5’-CCTCCTCCGAATGAATGA AA-3’; Reverse primer: 5’-TCCGATACTGGAAACG GAAG-3’. Primers for E02H9.5 are: Forward primer: 5’-ATAGGCAATATTTTGTTAGT-3’; Reverse primer: 5’-GCTGAAGCCGCGGCCATTCA-3’. Primers for C50F7.10 are: Forward primer : 5’-GATATTCTGACC TCTTACAG-3’; Reverse primer : : 5’-ATCCCAAGT ACTGAATCCGC -3’. To avoid cross-amplification of RNA sequence present in RNAi-fed worms, both E02H9.5 and C50F7.10 specific forward primers were designed to hybridize the 5’-UTR region not included in the RNAi-targeted sequences. The corresponding reverse primers were designed to hybridize the boundary of the first and the second exons in order to prevent cross-amplification of possible contaminating genomic DNA. The RNA level of each gene of interest was normalized to the tba-2 reference gene level for comparison and results of three independent experiments were treated as described in (Pfaffl, 2001). As shown in Figure S1, each construct was able to induce a similar knockdown of both C50F7.10 and E02H9.5 gene expression. Thus, in this study, the knockdown of both paralogues, indifferently induced by either construct, will be referred as *klotho* RNAi and the product of either *C. elegans* gene will be named Klotho.

Clr phenotype suppression assay.Clr-1-suppressing activity was assayed in a temperature-sensitive clr-1(e1745) background. After 4 days of RNAi treatment at 15°C, adult worms were either maintained at the permissive temperature (15°C) or shifted to the restrictive temperature (25°C), then examined 24h later for the suppression of the Clr phenotype. At least 50 worms were examined per plate and results are representative of three independent experiments. Worms were photographed under bright-field illumination.

### Oxidative stress resistance assays

Menadione was used as a potent oxidative stress inducer, as previously described [45]. After 4 days of RNAi treatment at 20°C, adult worms from different genetic backgrounds were transferred onto 24-well plates (about 30 worms per well) containing S complete medium supplemented with 25 mM of Menadione (Sigma-Aldrich) and transformed HT115 bacteria to maintain the RNAi treatment during the assay. Controls were performed in the absence of Menadione. At least four independent experiments were carried out for 72h at 20°C and the viability of worms was scored, using 1 μM SYTOX Green Nucleic Acid Stain (Molecular probes) to discriminate fluorescent dead worms. Survival histograms were analyzed with XLSTAT software (Addinsoft, New York, NY, USA), using the Student's-t-test at *p < 0.05 signification level.

## SUPPLEMENTAL FIGURE

Figure S1. Targeting for klotho gene expression by RNAi. N2 adult worms were submitted to RNAi feeding for either C50F7.10 or E02H9.5 gene knockdown. The relative mRNA levels for each gene were quantified using LightCycler software (Roche Diagnostics). After normalization to the mRNA level of tubulin (TBA2), results were treated as described in [59] and expressed as mean + standard error of the mean (SEM) from three independent experiments.
