# Supplementary figures and images for: Klotho interferes with a novel FGF-signalling pathway and insulin/Igf-like signalling to improve longevity and stress resistance in Caenorhabditis elegans
Source: Aging (Albany NY). 2010 Sep 9;2(9):567–81. doi: 10.18632/aging.100195 (PMC2984606; doi:10.18632/aging.100195)

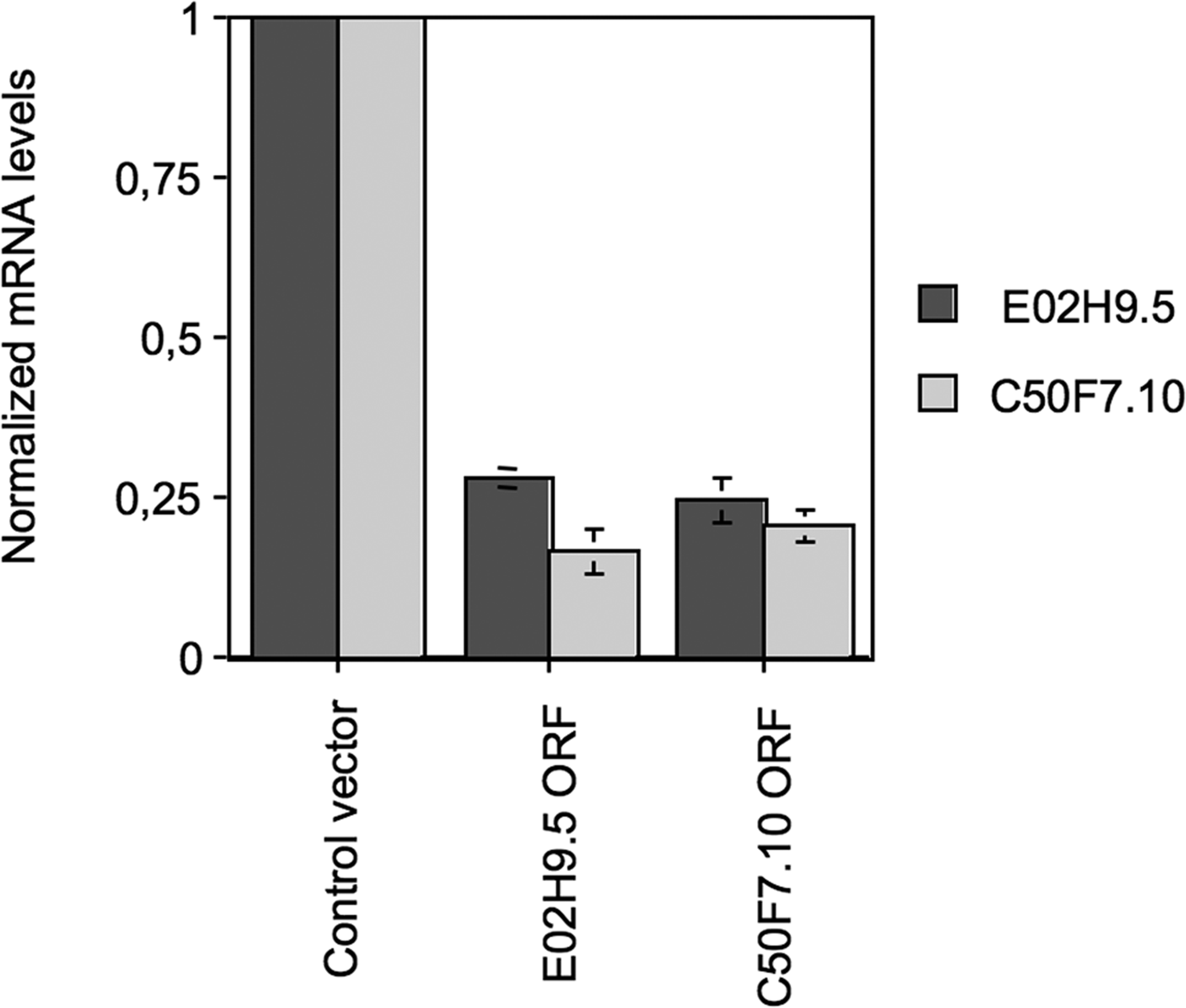

Supplement: Figure S1. — N2 adult worms were submitted to RNAi feeding for either C50F7.10 or E02H9.5 gene knockdown. The relative mRNA levels for each gene were quantified using LightCycler software (Roche Diagnostics). After normalization to the mRNA level of tubulin (TBA2), results were treated as described in [59] and expressed as mean + standard error of the mean (SEM) from three independent experiments. [file aging-02-567-s001.tif]
